# Case report: A panorama gene profile of ovarian cancer metastasized to axillary lymph node

**DOI:** 10.3389/fimmu.2025.1548102

**Published:** 2025-01-24

**Authors:** Yu Xia, Yu Huang, Zheng Liu, Siyuan Song, Yi Wang, Jing Luo

**Affiliations:** ^1^ School of Medicine, University of Electronic Science and Technology of China, Chengdu, China; ^2^ Department of Obstetrics and Gynecology, Sichuan Provincial People’s Hospital, University of Electronic Science and Technology of China, Chengdu, China; ^3^ Department of Pathology, The University of Texas MD Anderson Cancer Center, Pathology, Houston, TX, United States; ^4^ Department of Neuroscience, Baylor College of Medicine, Houston, TX, United States; ^5^ Department of Critical Care Medicine, Sichuan Provincial People’s Hospital, University of Electronic Science and Technology of China, Chengdu, China; ^6^ Clinical Immunology Key Laboratory of Sichuan Province, Sichuan Provincial People’s Hospital, Chengdu, China; ^7^ Department of Breast Surgery, Sichuan Provincial People’s Hospital, University of Electronic Science and Technology of China, Chengdu, China

**Keywords:** ovarian cancer, axillary lymph node, metastasis, BTK mutation, EPHA5 mutation, metabolism related mutations, drug resistance, pathway analysis

## Abstract

**Background:**

Ovarian cancer is among the most lethal gynecologic malignancies, with a high proportion of patients diagnosed at advanced stages, leading to poor survival outcomes. Axillary lymph node metastasis from ovarian cancer is extremely rare and the mechanism is still unclear.

**Methods:**

A comprehensive set of clinical and gynecologic oncology assessments were performed, including ultrasound, mammography, MRI, transvaginal ultrasound, and tissue staining. To unravel the carcinogenesis, the next-generation sequencing (NGS) was performed.

**Results:**

Conventional examinations and imaging suggested the presence of both occult breast cancer and ovarian cancer. However, immunohistochemical staining confirmed the diagnosis of high-grade serous ovarian carcinoma. Further analysis of NGS identified two novel missense mutations, D326E in BTK (Bruton’s tyrosine kinase) at SH2 domain and D251E in EPHA5 (EPH receptor A5), along with other known cancer- associated mutations. These mutations, particularly the novel missense mutations, may lead to metastasis to the axillary lymph nodes and drug resistance. Therefore, based on these findings, the chemotherapy regimen was adjusted accordingly.

**Conclusion:**

This is the first report on the panorama gene profile of ovarian cancer metastasis to axillary lymph node and we found two novel mutations (BTK pD326E and EPHA5 pD251E). This study unraveled the potential mechanism of genetic mutation for tumor metabolism, drug resistance, and metastasis.

## 1 Introduction

Ovarian cancer is considered to be one of the most lethal gynecologic malignancies, with many patients presenting with advanced-stage disease at diagnosis (1). Unlike many other cancers, ovarian cancer typically spreads within the peritoneal cavity rather than through hematogenous routes. The most common sites of metastasis are pelvic and para-aortic lymph nodes (2). However, axillary lymph node metastasis is extremely rare, which may lead to misdiagnosis or miss-diagnosis by physicians or surgeons. To date, there are only two cases of breast cancer metastasis to ovarian cancer (3, 4). However, only one report assessed two breast cancer-sensitive genes as *BRCA1* and *BRCA2* mutations, and they failed to find the mutations of these two genes (4). Therefore, a comprehensive analysis of cancer hub gene (5, 6) (including *KRAS*, *TP53*, and so on), breast sensitive genes (7) (such as *BRCA1*, and *BRCA2*) is of great need.

In this study, we began with a thorough physical examination and tissue staining analysis of the axillary lymph node and ovarian cancer. Following this, we employed next-generation sequencing (NGS) to identify mutations associated with key biological pathways. These included nucleotide metabolism pathways (e.g., thymine catabolism, thymidine *de novo* biosynthesis, and homocysteine metabolism), DNA repair pathways (e.g., nucleotide excision repair and single-strand break repair), as well as pathways linked to drug resistance, homologous recombination deficiency (HRD), and microsatellite stability.

Among these mutation profiles, D326E is a novel missense mutation for *BTK*, Bruton’s tyrosine kinase (a nonreceptor kinase) has been reported to play a crucial role in B cell receptor (BCR) signaling. Mutations or increased expression of *BTK* are correlated in many types of B cell-derived malignancy, including chronic lymphocytic leukemia (CLL) and other B cell cancers (8). Importantly, elevated expression is not only correlated with B-cell related carcinoma. Solid tumors as breast cancer (9), pancreatic cancer (10) and other types of cancer are directly or indirectly mediated by aberrant *BTK* expression or mutations. Therefore, targeting C483S of *BTK*, the most common *BTK* mutation, has led to the development of covalent (irreversible) inhibitor and its clinical application (11). However, due to the significant role of BCR for normal B cell development and adaptive immunity, covalent (ibrutinib, acalabrutinib, and zanubrutinib) and noncovalent (pirtobrutinib and nemtabrutinib, ongoing clinical investigation) BTK inhibitor, raises drug resistance correlated with site mutations as L528W, Y223F, which is well documented elsewhere (12–14). Of note, no matter whether covalent or noncovalent BTK inhibitors target these documented mutations, combinational application of BTK inhibitor is needed to overcome BTK mutations, such as V416L, A428D, M437R, K430R and T474I, and these mutations has been experimentally demonstrated to be correlated with autophosphorylation or kinase- inactivation (12, 15). Similarly, the *EPHA5* (EPH family A5) belongs to the ephrin receptor subfamily of the protein-tyrosine kinase family. The binding of EPH and its receptor is correlated with the development, angiogenesis, and cancer (16, 17). Accumulating evidence reveals that *EPHA5* mutations contribute to tumor immunity, tumor migration and invasion (18–20). Therefore, it is of urgent need to find novel mutation sites of *BTK* or *EPHA5* for drug development for multiple B-cell cancers and other solid tumors.

Overall, this is the first comprehensive report of an ovarian cancer patient with initial axillary lymph node metastasis, combining traditional technology with advanced genome wide sequencing to profile cancer hub gene and related signaling pathway mutations. Importantly, we found two novel gene mutation sites, which provide new strategy for oncotherapy.

## 2 Materials and methods

### 2.1 Patient information

A 56-year-old female was admitted to Sichuan Provincial People’s Hospital with a chief symptom of a right axillary mass for one month. The patient provided written informed consent for all diagnostic procedures and treatments. This study was conducted in accordance with the ethical standards of the Sichuan Provincial People’s Hospital’s Institutional Review Board and adhered to the principles outlined in the Declaration of Helsinki. The patient’s social history revealed that she was a non-smoker and denied alcohol use. Psychosocially, the patient reported experiencing significant stress due to her illness and limited familial support.

### 2.2 Clinical examination

On physical examination, the patient presented with a firm, poorly mobile lymph node mass in the right axilla, approximately 4.0 cm × 3.5 cm. The overlying skin was intact, and no other abnormalities were noted on the clinical examination.

### 2.3 Diagnostic imaging

#### 2.3.1 Gynecological Ultrasound

A transvaginal ultrasound was performed using the Philips EPIQ 7 ultrasound system. It revealed a cystic mass in the right adnexal area categorized as O-RADS 3, with pelvic-abdominal fluid noted. A slightly enlarged uterus was also observed.

#### 2.3.2 Mammography

Bilateral mammograms were obtained using a Hologic Selenia Dimensions system, revealing no significant masses in the breast tissue. However, the right axilla demonstrated enlarged lymph nodes, raising suspicion of metastatic disease.

#### 2.3.3 Breast MRI

MRI was performed on a Siemens Magnetom Skyra 3T scanner. Multiple enlarged lymph nodes with scattered enhancing nodules were observed in the right axilla. Additionally, parenchymal enhancement in both breasts was noted, but no definitive primary breast lesion was identified. This supported the suspicion of metastasis from an extramammary primary tumor.

#### 2.3.4 Enhanced Abdominal CT

CT imaging was conducted using a Siemens SOMATOM Force system. The scan revealed bilateral adnexal masses, with irregular enhancement of the peritoneum, omental nodularity, and ascites suggestive of peritoneal carcinomatosis. No evidence of primary breast cancer was observed.

### 2.4 Histopathological examination of axillary lymph node

Biopsies from the right axillary lymph node were collected and analyzed. Tissue sections were stained with hematoxylin and eosin (data not shown). Immunohistochemical staining was performed using the Ventana Benchmark Ultra automated system (Roche Diagnostics). Immunohistochemical staining was performed using a panel of markers to determine the origin of the metastatic cells. The following primary antibodies were applied: CK7 (1:200, Abcam), ER (1:100, Abcam), GATA3 (1:150, Abcam), Ki-67 (1:300, Abcam), p53 (1:200, Abcam), PAX8 (1:100, Abcam), PR (1:150, Abcam), WT-1 (1:200, Abcam), CK (1:100, Abcam), p16 (1:200, Abcam), MLH-1 (1:150, Abcam), MSH-2 (1:200, Abcam), MSH-6 (1:200, Abcam), and PMS2 (1:200, Abcam). After incubation with the primary antibodies, sections were treated with a biotinylated secondary antibody (Goat Anti-Rabbit or Goat Anti-Mouse IgG, 1:500, Vector Laboratories). Detection was performed using an avidin-biotin complex (ABC) method (VECTASTAIN^®^ ABC Kit, Vector Laboratories), and DAB (3,3′- diaminobenzidine) was used as the chromogen (DAB Substrate Kit, Vector Laboratories). Hematoxylin (Sigma-Aldrich) was applied as a counterstain. This tissue staining profile was used to assess the potential origin of the metastatic cells. The pattern of immunoreactivity supported a diagnosis of high-grade serous ovarian carcinoma metastasizing to the axillary lymph nodes.

### 2.5 Next-generation sequencing

The tumor genomic analysis was performed using targeted region capture high- throughput sequencing. A panel of 437 cancer-related genes was examined, with a focus on microsatellite instability (MSI), tumor mutational burden (TMB), and homologous recombination deficiency (HRD) status. RNA and DNA were extracted from formalin- fixed paraffin-embedded (FFPE) tumor samples using the Qiagen AllPrep DNA/RNA Mini Kit (Qiagen, Hilden, Germany) following the manufacturer’s protocol. Library preparation was performed using the KAPA HyperPrep Kit (Kapa Biosystems, Wilmington, MA, USA) with indexed adapters to allow multiplexing. Sequencing was carried out on the Illumina NovaSeq 6000 platform (Illumina, San Diego, CA, USA), which provided high-throughput, paired-end sequencing with a read length of 150 base pairs. The analysis covered single nucleotide variations (SNVs), small insertions and deletions (indels), gene fusions, and copy number variations (CNVs) across the targeted regions. The raw sequencing data were processed using the Illumina DRAGEN Bio-IT Platform for variant calling, alignment, and quality control. Identified somatic mutations, including point mutations and structural variants, were cross-referenced against established cancer mutation databases, such as COSMIC and ClinVar, to confirm oncogenic significance.

### 2.6 Gynecological surgery

The patient was informed that the current appropriate surgical method is total hysterectomy, double adnexectomy, appendectomy, omentectomy, pelvic and abdominal lesion resection. However, the patient refused to undergo extensive resection and only agreed to perform adnexectomy. Therefore, the surgery group performed laparoscopic right adnexectomy and abdominal wall peritoneal biopsy. For the anesthesia of the patient, it is a combination of intravenous clopofol injection (20 mg pump), 1.5% sevoflurane for inhalation, and remifentanil hydrochloride (0.1ug/kg/min pump for injection).

On April 20, 2024, the patient took the bladder lithotomy position on the disinfected towel, established pneumoperitoneum, and inserted the Trocar; Then, took the head and feet low, exposed the pelvis. Separated the right pelvic vestibular ligament, bipolar coagulation, bipolar coagulation along the mesangium of the fallopian tube, bipolar coagulation of the ovary propria ligament, and the fallopian tube to the uterine angle. Took the peritoneal nodule of the abdominal wall, and took out the specimen completely for histological examination. Lastly, flush the pelvis, suck up the irrigation solution, and there is no obvious oozing serum point, the gauze instruments are correct, and the puncture hole is sutured.

During the operation, a small amount of yellowish fluid (approximately 300 m1) can be observed in the pelvis. Miliary nodules can be seen on the pelvic abdominal wall, liver surface, diaphragm surface, uterus and bilateral appendages, which are hard and brittle to bleed. A floral nodule of about 5 cm × 5 cm can be observed in the rectal uterine pit, and a large amount of inflammatory membranous material can be seen on the surface. A thin wall of about 3 cm × 3 cm mass was visible in the right fallopian tube, a gravelly nodule was visible on the surface of the ovary, and there was no obvious abnormality in the appearance of the left adnexa except for the miliary nodule. Miliary knots were visible on the surface of the appendix, and there was no obvious abnormality in the appearance of the omentum and small mesentery.

### 2.7 Histopathological examination of right abdominal lesion

Biopsies from the right abdominal lesion were collected and analyzed. Tissue sections were stained with hematoxylin and eosin (data not shown). Immunohistochemical staining was performed as previously addressed in this article.

### 2.8 Statistical analysis

The captured regions totaled 1.53 Mb across the exons of the specified genes, covering hotspot mutations, as well as regions associated with key cancer pathways. The microarray data was used to assess tumor mutational burden (TMB) by calculating the number of nonsynonymous mutations per megabase of the tumor genome. Homologous recombination deficiency (HRD) was evaluated through structural rearrangement analysis, including large-scale chromosomal aberrations. All sequencing was performed using high-throughput Illumina platforms with a minimum coverage depth sufficient to detect low-frequency variants. Descriptive statistics were used to summarize the mutation data. For significance testing, p-values below 0.05 were considered statistically significant.

## 3 Results

### 3.1 Patient presentation and diagnosis

**Figure 1:**
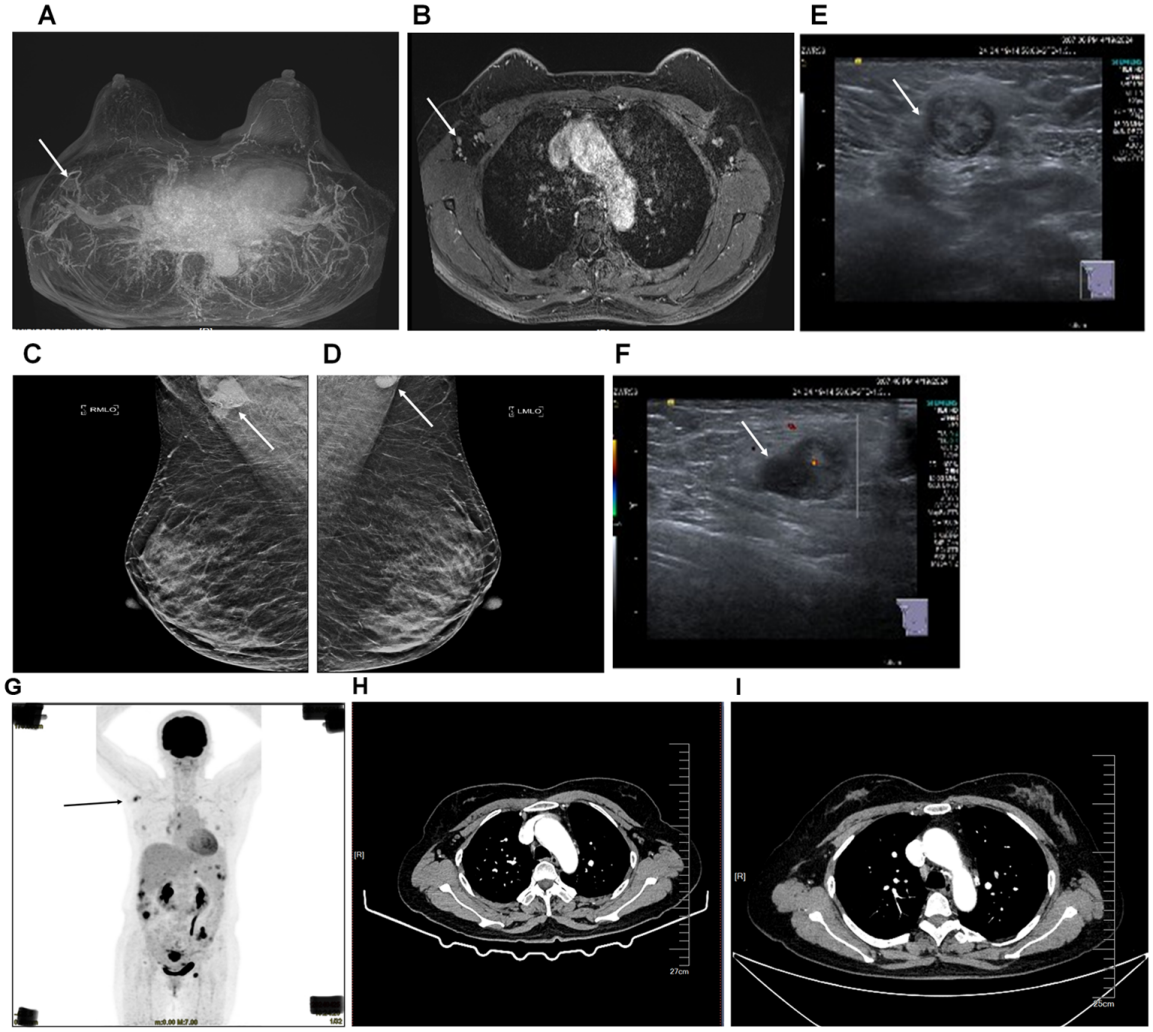
Imaging diagnosis of breast cancer and diagnostic imaging of axillary lymph node and ovarian tumor before and after surgery. **(A, B)**, MRI of the patient’s breast. **(C, D)**, mammography of the patient’s breasts. **(E, F)**, breast ultrasound of the patient’s lymph node. The arrow pointed at the enlarged lymph node in the right axilla. **(G)** PET-CT scan showing FDG-avid nodules throughout the body (black arrow), with significant uptake in the axillary lymph node and right axilla. **(H)** Enhanced chest CT scanning of the patient before surgery. The arrow pointed at the lymph nodes in the right axilla. **(I)** Enhanced chest CT scanning of the patient 4 months after surgery. The arrow pointed at the lymph node in the right axilla.

**Figure 2:**
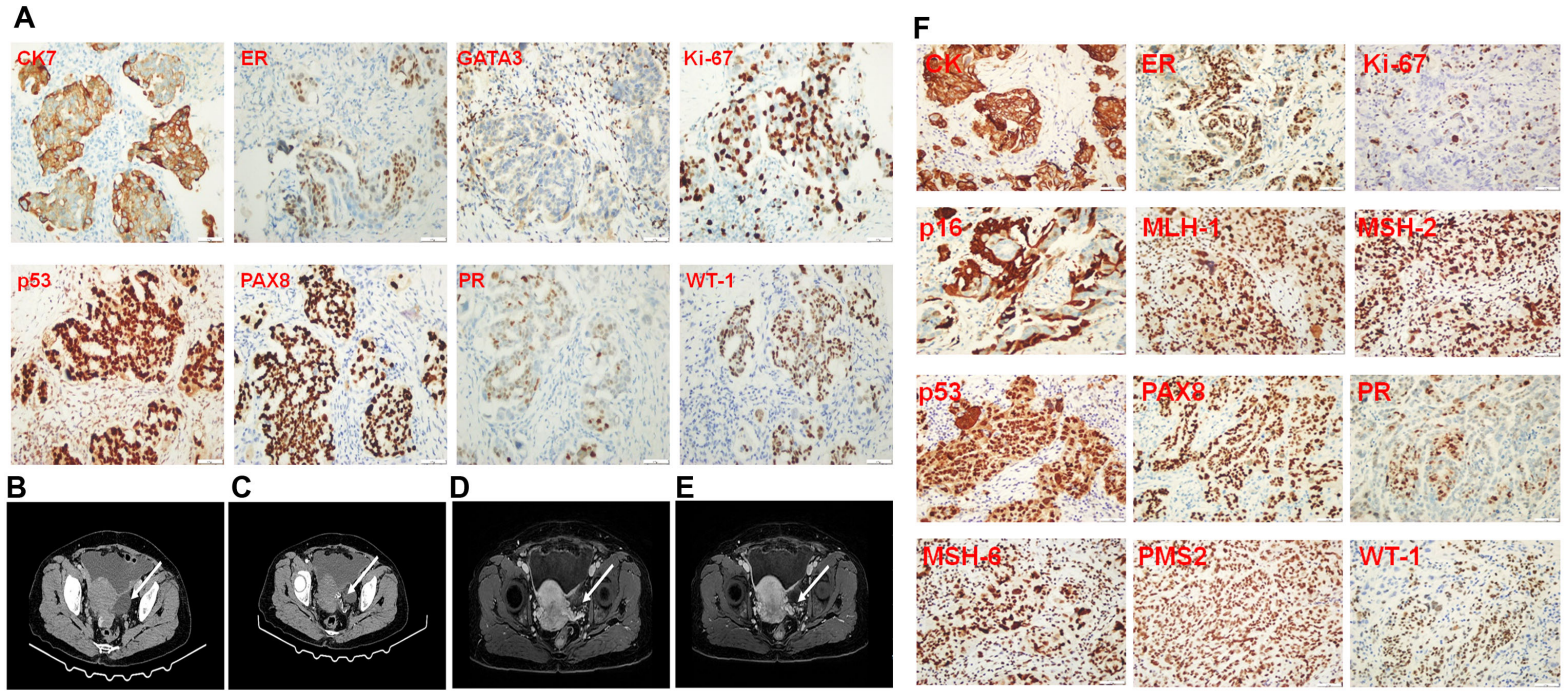
Immunopathological staining of a biopsy from the lymph node in the right axilla, abdomen imaging diagnosis, and immunopathological staining of ovarian cancer tissue resected during surgery. **(A)** Immunopathological staining of biopsy from the lymph node in the right axilla. **(B, C)**, CT scanning of the patient’s abdomen. **(D, E)**, MRI scanning of the patient’s abdomen. The arrow pointed at the enlarged lymph node in the right axilla. **(F)** Immunopathological staining of the ovarian cancer tissue resected from the surgery.

The patient, a 56-year-old woman, was initially suspected of having occult breast cancer based on axillary lymph node metastasis. She received Breast cancer-related imaging diagnosis, including MRI of the patient’s breast (**Figures 1A, B**), mammography of the patient’s breasts (**Figures 1C, D**), breast ultrasound of the patient’s lymph node (**Figures 1E, F**), and chest CT (**Figures 1H, I**). However, there seems to be no tumor cell at the breast and we observed it might exist at the right axillary lymph node. Therefore, this patient performed PET-CT scan. The PET-CT scan (**Figure 1G**) demonstrated FDG-avid nodules throughout the body, indicating widespread metastasis. To answer the question of the tumor cell origin, we performed axillary lymph node biopsy, hematoxylin and eosin staining (data not shown) and IHC staining (**Figure 2**). Meanwhile, enhanced abdominal imaging, including CT scanning (**Figures 2B, C**), MRI scanning of the patient’s abdomen (**Figures 2D, E**). These image data showed bilateral adnexal masses, omental caking, and pelvic-peritoneal involvement, which pointed toward high-grade serous ovarian carcinoma. The biopsy sections stained with hematoxylin and eosin reveal characteristic features of high-grade serous carcinoma, including pleomorphic tumor cells and high mitotic activity (data now shown). Histopathological analysis (**Figure 2F**) confirmed the ovarian cancer infiltrated to the axillary lymph node. Immunohistochemical staining of the axillary lymph node (**Figure 2A**) and the ovarian tumor (**Figure 2F**) supported the diagnosis of metastasis from ovarian cancer. The axillary lymph node showed strong positivity for CK7, ER, and WT-1, alongside weak positivity for GATA3, indicative of a non-breast primary tumor. Other markers, including Ki-67, showed high proliferative activity. The ovarian tumor demonstrated a similar immune profile, further solidifying the connection between the two sites. WT-1, PAX-8, and p53 were also strongly positive, characteristic of high- grade serous ovarian carcinoma.

### 3.2 D326E of BTK missense mutation

**Figure 3:**
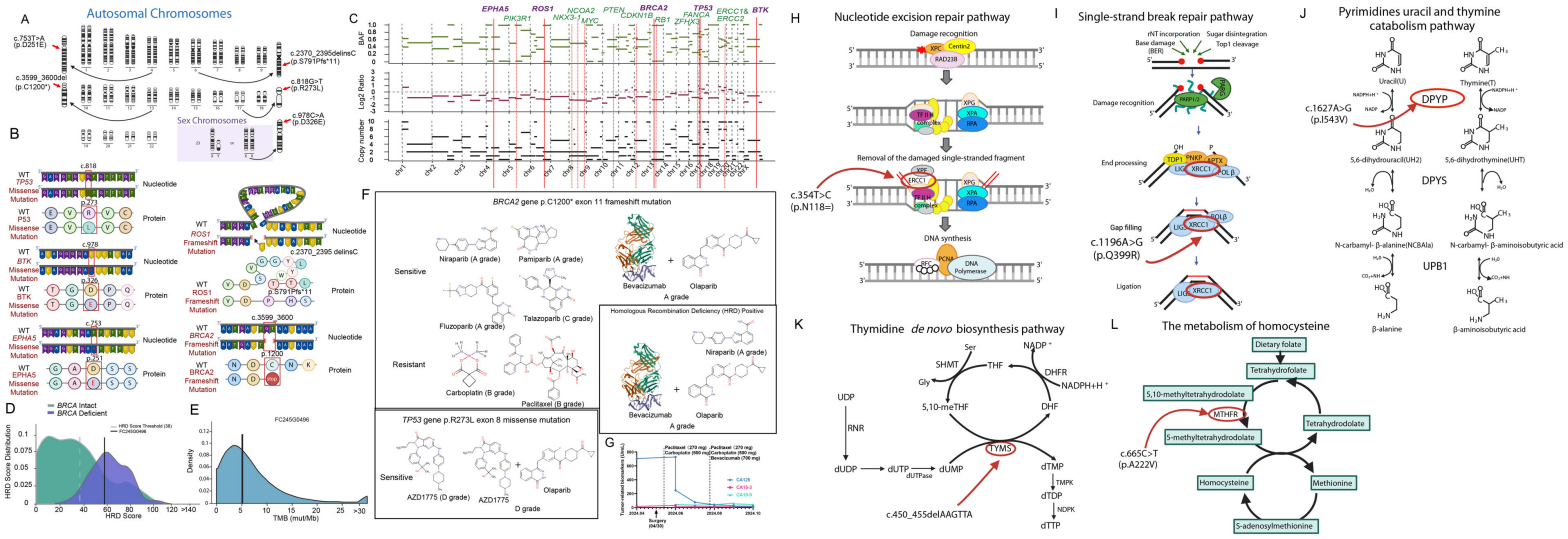
Tumor-sensitive gene mutations and drug sensitivity in ovarian cancer, along with mutations affecting drug metabolism and DNA repair pathways. **(A)** Chromosomal representation of the tumor showing mutations in key genes, including *BTK* and *EPHA5*. **(B)** Mutations of *BTK*, *EPHA5*, *ROS1*, *TP53* and *BRCA2* with diagrams illustrating nucleotide and protein changes. **(C)** Copy number variations (CNVs) and amplifications detected in cancer-associated genes. **(D)** HRD score distribution. **(E)** Tumor mutational burden (TMB) analysis. **(F)** Drug sensitivity analysis based on mutational profile, indicating potential sensitivity to PARP inhibitors and resistance to traditional chemotherapies like carboplatin and paclitaxel. **(G)** The patient received laparoscopic right adnexectomy on April 30th, 2024. Subsequently, she received TP therapy on May 16 (1st chemotherapy), June 3rd (2nd chemotherapy), June 25 (3rd chemotherapy). On the following treatment, this patient received both chemotherapy (paclitaxel plus carboplatin) and immunotherapy (bevacizumab) on July 19 (4th therapy), August 12 (5th therapy), September 3rd (6th therapy), September 25 (7th therapy) and October 17 (8th therapy). Tumor-related biomarkers as CA 125, CA 19-9 and CA- 15-3 was quantified before the surgery and after the drug therapy. **(H)** Nucleotide excision repair pathway highlighting the effect of the ERCC1 p.N118= mutation on DNA repair. **(I)** Pyrimidine catabolism pathway showing the impact of the DPYD p.I543V mutation on drug metabolism. **(J)** Single-strand break repair pathway affected by the XRCC1 p.Q399R mutation. **(K)** Thymidine *de novo* biosynthesis pathway showing the effect of the TYMS c.450_455delAAGTTA mutation. **(L)** Homocysteine metabolism pathway highlighting the impact of the MTHFR c.665C>T (p.A222V) mutation on folate metabolism.

Because ovarian cancer metastasizing to the right axillary lymph node is rare, we performed a panel of gene mutation profiles and tried to elucidate the mechanism of this metastasis. Shown in **Figure 3A**, a novel missense mutation in the *BTK* gene, located on chromosome X, specifically at position c.978C>A. This mutation occurs in exon 12 of the coding sequence, resulting in a substitution from cytosine to adenine (C>A), which changes the amino acid from aspartic acid (D) to glutamic acid (E), labeled as p.D326E (**Figure 3B**). This site mutation of the *BTK* leads to the mutation of BTK protein at the 326 site from aspartic acid to glutamic acid (D326E). Studies on other proteins revealed that the substitution of aspartic acid with glutamic acid may lead to increases in the unfolding transition temperature of a protein (21). Known for its role in B-cell receptor signaling, aberrant folded BTK may lead to failure of autophosphorylation and subsequent cell survival or immune evasion. Although several BTK mutations have been well documented, D326E has not been previously reported in ovarian or any other cancers. Importantly, this mutation is located at the SH2 domain (amino acid 280 to 377) of BTK. Studies on SH2 domain revealed that mutation at this domain may perturb the activity of BTK (22). The variant was detected with a frequency of 9.89% in the tumor cells, indicating a potentially significant impact on the tumor’s behavior (**Figures 3A, B**).

### 3.3 D251E of EPHA5 missense mutation

Another novel mutation, D251E of *EPHA5*, was identified, located on chromosome 7 at position c.753T>A. This mutation occurs in the extracellular ligand-binding domain of EPHA5, a key receptor in the ephrin receptor family (**Figures 3A, B**). EPHA5 is involved in critical processes such as cellular communication, adhesion, and migration.

Alterations in this gene can disrupt proper protein folding and membrane localization, impairing its function as a transmembrane receptor, which may be significant in the context of cancer (23, 24).

The D251E mutation results in an amino acid change from aspartic acid (D) to glutamic acid (E). This substitution is predicted to impact the receptor’s structure, likely affecting its ability to interact with ligands and communicate with adjacent cells. Such structural changes could weaken intercellular adhesion, facilitating cancer cell detachment and migration, thus promoting metastatic behavior. In ovarian cancer, specifically, disruptions in EPHA5 signaling are linked to enhanced tumor invasiveness, possibly aiding immune evasion and metastasis. This mutation was detected in 17.55% of the tumor genome in the current study, underscoring its potential role in tumor progression.

### 3.4 Tumor suppressive gene mutations

In this study, we conducted a comprehensive panel of gene mutations, identifying critical variants beyond BTK and EPHA5. These mutations include TP53 c.818G>T (p.R273L), ROS1 c.2370_2395delinsC (p.S791Pfs11), and BRCA2 c.3599_3600del (p.C1200*), each of which significantly impacts protein function and contributes to tumor behavior and therapeutic resistance (**Figures 3B, C**).

#### 3.4.1 TP53 c.818G>T Missense Mutation

This mutation occurs on chromosome 17, specifically at nucleotide position c.818G>T within exon 7 of the TP53 gene. It results in an amino acid change from arginine (R) to leucine (L) at position 273, within the DNA-binding domain of the p53 protein. This domain is essential for DNA repair and apoptosis, key functions of the p53 tumor suppressor. The R273L mutation is a “hotspot” alteration that compromises p53’s DNA-binding ability, leading to a loss of tumor-suppressive function. Consequently, this mutation contributes to genomic instability and is associated with chemoresistance and poor prognosis in various cancers.

#### 3.4.2 ROS1 c.2370_2395delinsC Frameshift Mutation

This frameshift mutation is located on chromosome 6 at c.2370_2395delinsC within exon 11 of the ROS1 gene. It causes a shift in the reading frame starting at amino acid 791, converting serine (S) to proline (P) and introducing a premature stop codon after 11 altered amino acids. This truncation results in the loss of the kinase domain, which is crucial for ROS1’s role in cell proliferation and survival signaling pathways. The absence of kinase activity due to this truncation likely promotes unregulated cell growth, increasing the tumorigenic potential of the cancer cells.

#### 3.4.3 BRCA2 c.3599_3600del Frameshift Mutation

The BRCA2 gene contains a frameshift mutation at chromosome 13 position c.3599_3600del in exon 11, which introduces a premature stop codon, truncating the BRCA2 protein at amino acid 1200. This results in the loss of crucial C-terminal domains necessary for DNA repair via homologous recombination. As a tumor suppressor, BRCA2 plays a vital role in DNA damage response (DDR), ensuring genomic stability. The absence of these domains compromises the protein’s stability and increases degradation susceptibility, undermining its tumor-suppressive function. This mutation creates homologous recombination deficiency (HRD), rendering the tumor sensitive to PARP inhibitors like olaparib and niraparib, which target HRD in cancer cells for effective therapeutic intervention.

### 3.5 Structural and copy number variations

Chromosomal alterations and copy number variations (CNVs) were observed across several key genes involved in cancer progression. As shown in **Figure 2C**, amplifications were detected in genes such as PIK3R1, ROS1, and EPHA5, all of which are linked to cell survival and proliferation pathways. Losses in tumor suppressor genes such as RB1 and CDKN1B further highlight the genomic instability of this tumor (**Figure 3C**).

### 3.6 Homologous recombination deficiency and tumor mutational burden

HRD scoring and TMB analysis were conducted to assess the tumor’s genomic instability and potential sensitivity to targeted therapies. The patient was classified as HRD-deficient (**Figure 3D**), with an elevated HRD score, suggesting a higher likelihood of benefiting from PARP inhibitors. Additionally, the tumor mutational burden (TMB) was moderate, as depicted in **Figure 3E**, supporting the potential for immunotherapy strategies in the future.

### 3.7 Drug sensitivity analysis

The **Figure 3F** panel highlights the predicted sensitivity and resistance to various chemotherapeutic agents based on the patient’s mutational profile. BRCA2 mutations indicate sensitivity to PARP inhibitors, including Niraparib and Olaparib (A grade). However, mutations in TP53 are associated with resistance to some conventional therapies such as Carboplatin and Paclitaxel, necessitating consideration of alternative treatments.

### 3.8 Tumor marker response

The patient’s treatment response, tracked through tumor marker levels, is shown in **Figure 3G**. Following treatment with Paclitaxel liposome, Carboplatin, and Bevacizumab, there was a significant reduction in tumor markers (CA125, CA15-3, CA19-9), although concerns for recurrence emerged based on rising levels over time.

### 3.9 Mutations related to metabolism and repair

Next-generation sequencing identified additional somatic mutations in key genes that affect metabolism, DNA repair, and cellular metabolism pathways (**Figures 3H–L**). These include mutations in the DPYD, ERCC1, MTHFR, TYMS, and XRCC1 genes, which may influence the patient’s response to chemotherapy and overall prognosis.

#### 3.9.1 ERCC1 p.N118N Mutation

This gene encodes a protein involved in the nucleotide excision repair pathway, critical for resolving DNA damage caused by chemotherapy agents like platinum-based drugs (**Figure 3H**). The mutation identified here may reduce the efficacy of platinum-based therapies such as carboplatin. Given its role in repairing DNA crosslinks, any deficiencies in ERCC1 could increase the cancer cells’ sensitivity to DNA-damaging agents but also raise the risk of resistance through alternative repair mechanisms.

#### 3.9.2 XRCC1 p.Q399R Mutation

XRCC1 plays an important role in the base excision repair (BER) pathway, responsible for fixing single-strand breaks in DNA caused by reactive oxygen species and chemotherapy-induced damage (**Figure 3I**). Mutations in XRCC1 can compromise the repair process, making cells more vulnerable to DNA damage and increasing the efficacy of certain chemotherapeutic agents.

#### 3.9.3 DPYP p.I543V Mutation

DPYP encodes dihydropyrimidine dehydrogenase, a key enzyme in the breakdown of pyrimidine bases such as uracil and thymine, and is involved in the catabolism of fluoropyrimidine drugs (**Figure 3J**). The mutation found could potentially alter the patient’s ability to metabolize 5-fluorouracil, commonly used in ovarian cancer treatment, leading to altered drug efficacy or increased toxicity.

#### 3.9.4 TYMS c.450_455delAAGTTA Mutation

TYMS encodes thymidylate synthase, an enzyme critical in the *de novo* synthesis of thymidine (**Figure 3K**). This deletion could potentially result in impaired DNA synthesis, affecting cell replication and influencing the tumor’s sensitivity to drugs targeting the folate pathway, such as methotrexate.

#### 3.9.5 MTHFR c.665C>T (p.A222V) Mutation

The MTHFR gene is responsible for converting 5,10-methylenetetrahydrofolate to 5-methyltetrahydrofolate, a key step in the folate metabolism pathway (**Figure 3L**). The mutation could impact the patient’s ability to metabolize folate, influencing their response to folate-dependent chemotherapeutic agents like methotrexate. It could also raise homocysteine levels, increasing the risk of thromboembolic events, a common complication in ovarian cancer patients.

### 3.10 Gene pathway involvement and mutational impact

**Figure 4:**
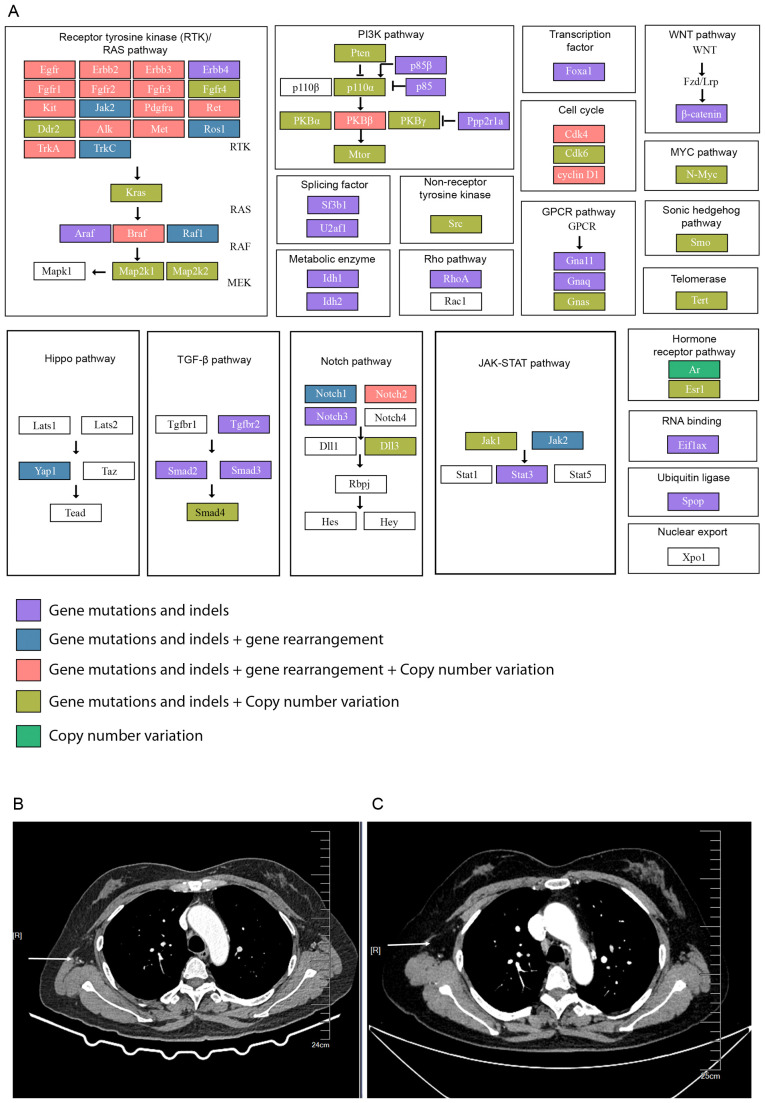
Mutations in cancer-associated signaling pathways and enhanced chest CT scans of the patient after surgery. **(A)** A comprehensive overview of the key signaling pathways affected by the patient’s mutational profile, including: (1) Receptor Tyrosine Kinase (RTK)/RAS Pathway: Mutations in EGFR, ROS1, and other tyrosine kinases activating downstream signaling pathways promoting tumor growth and metastasis. (2) PI3K/AKT Pathway: Alterations in PIK3R1 and AKT2 leading to enhanced cell survival and drug resistance. (3) JAK-STAT Pathway: Mutations in JAK1 and JAK2 disrupting immune signaling and promoting immune evasion. (4) WNT, MYC, and Cell Cycle Pathways: Mutations leading to uncontrolled cell proliferation and epithelial-mesenchymal transition (EMT), promoting metastasis. **(B)** 2 months after surgery and **(C)** 4 months after surgery. The arrow pointed at the lymph nodes in the right axilla.

We provide a comprehensive view of the altered signaling pathways in the patient’s tumor, driven by the mutations identified in genes such as TP53, BTK, EPHA5, and ROS1 (**Figure 4A**). The pathways affected include:

#### 3.10.1 Receptor Tyrosine Kinase (RTK)/RAS Pathway

This pathway is heavily mutated, with alterations in several genes like FGFR1, EGFR, and ROS1. These mutations are known to activate downstream signaling, leading to uncontrolled cell growth and survival. In the case of ROS1 mutations, there is evidence of its involvement in cancer progression through receptor tyrosine kinase signaling, promoting metastasis and chemoresistance.

#### 3.10.2 PI3K/AKT Pathway

Alterations in genes such as PIK3R1 and AKT2 suggest hyperactivation of the PI3K/AKT pathway, which plays a critical role in promoting cancer cell survival, proliferation, and drug resistance. This pathway is particularly important in ovarian cancer, where mutations in PTEN and other related genes contribute to tumor aggressiveness.

#### 3.10.3 JAK-STAT Pathway

The JAK-STAT pathway, involved in immune regulation and cytokine signaling, is implicated in promoting tumor growth and immune evasion. Mutations in genes like JAK1 and JAK2 may disrupt normal immune responses, contributing to the tumor’s ability to metastasize and resist therapies.

#### 3.10.4 WNT, MYC, and Cell Cycle Pathways

Dysregulation of these pathways due to mutations in transcription factors such as MYCN and cell cycle regulators like CDK4 leads to aberrant cell proliferation. The WNT pathway is associated with epithelial- mesenchymal transition (EMT), a process that is crucial for metastasis.

### 3.11 Treatment and prognosis

Based on the genomic sequencing data, the therapy of the patients after surgery is TP chemotherapy (paclitaxel liposome 270 mg ivgtt d1 plus carboplatin 500 mg ivgtt d1, q21 d) initially on the first three periods (May 16, June 3rd, and June 25). Later bevacizumab (700 mg) immunotherapy was supplemented with TP chemotherapy, starting from July 19 till now. Patients are regularly rechecked for tumor markers (CA125, CA15-3, and CA19-9), and a declining trend was observed within 4 months after treatment (**Figure 1G**). This patient’s lymph node mass was decreased in 2 months and 4 months after therapy (**Figures 4B, C**). However, as this patient was admitted to the hospital in the past six months, further follow-up studies are needed.

## 4 Discussion

This study presents three significant innovations. First, the patient’s initial presentation with right axillary lymph node metastasis as the primary finding is an uncommon feature in ovarian cancer. Typically, ovarian cancer metastasizes through peritoneal spread or direct implantation within the abdominal cavity, with common sites including the peritoneum, omentum, and diaphragm (25). The rare involvement of a distant site such as the right axillary lymph node challenges the conventional understanding of metastatic pathways in ovarian cancer. Second, the mechanism of lymphatic or hematogenous spread leading to right-sided lymph node involvement suggests a potential cross-talk between the bloodstream and the lymphatic system, a phenomenon not frequently explored in this context. This points to a novel pathway that may involve tumor cells circulating through blood vessels and selectively seeding in regional lymphatics, indicating a complex interplay that could redefine metastatic behavior in high-grade serous ovarian carcinoma. Third, we performed a panorama gene profile. We discovered not only the common mutations including the tumor suppressive genes, metabolism-related genes, and DNA repair genes, but also the unique mutations in the BTK (D326E) and EPHA5 (D251E) genes adds further novelty, as these mutations have not been reported in ovarian cancer previously. This highlights potential genetic contributors to lymphatic metastasis and could pave the way for new diagnostic and therapeutic considerations.

While axillary lymph node metastasis from ovarian cancer is rare, our case represents the first report of BTK pD326E and EPHA5 pD251E mutations contributing to such metastasis. These novel mutations have not been previously reported in ovarian cancer, and their discovery may help elucidate the mechanisms underlying lymphatic metastasis and chemoresistance in this malignancy. Comprehensive analysis, including conventional imaging and immunohistochemistry, pointed to a diagnosis of high-grade serous ovarian carcinoma, which was further substantiated by next-generation sequencing that uncovered these unique mutations.

BTK (Bruton’s tyrosine kinase) is primarily known for its role in B-cell development and signaling, particularly in hematologic malignancies (26–28). BTK’s kinase activity involves phosphorylation of substrates like PLCγ2 (12, 29) which plays a role in calcium signaling and cell survival. The pD326E mutation identified in our study may affect BTK’s kinase function, though whether it leads to loss or gain of activity remains unclear. In other BTK mutations, loss of kinase activity has been observed, leading to defects in B-cell function. However, mutations affecting kinase activation sites can also promote aberrant signaling, as seen in cancers. Further research is needed to determine whether BTK inhibitors, already approved for B-cell malignancies, could provide a novel therapeutic approach for ovarian cancer patients harboring similar mutations.

EPHA5, a member of the ephrin receptor family, plays a critical role in mediating cell adhesion and migration through its binding to ephrin-A ligands (30, 31). The pD251E mutation, located in the extracellular domain, may impair ligand binding, potentially enhancing the metastatic capacity of ovarian cancer cells by disrupting normal cell communication. Activation of EPHA5 occurs via ligand-induced dimerization or proteolytic cleavage, initiating downstream pathways like PI3K/AKT and MAPK. Key proteins involved in this pathway include SRC, RHOA, and RAC1 (30, 32) which drive cytoskeletal changes and metastatic behavior. These alterations suggest that targeting EPHA5 signaling could offer therapeutic potential in metastatic ovarian cancer.

In addition to BTK and EPHA5, this patient harbored mutations in ROS1, BRCA2, and TP53, each of which plays a critical role in cancer progression and treatment resistance. The BRCA2 p.C1200 mutation, linked to homologous recombination deficiency (HRD), suggests that the patient may respond to PARP inhibitors (33, 34), which target cells deficient in DNA repair. Conversely, the TP53 p.R273L mutation is associated with chemoresistance, particularly to platinum-based therapies such as carboplatin. This highlights the importance of a personalized therapeutic approach that considers the combined effect of multiple mutations on the patient’s response to treatment.

The analysis of structural variations and copy number changes revealed genomic instability in several cancer-related pathways, including the RTK/RAS and PI3K/AKT pathways. These pathways are critical for cell growth and survival, and their dysregulation is a hallmark of many cancers, including ovarian cancer. The activation of these pathways through mutations in genes like PIK3R1 and ROS1 further complicates treatment options, as these alterations often confer resistance to conventional chemotherapy. The integration of pathway analysis into the patient’s genetic profile provides valuable insight into potential therapeutic targets, such as inhibitors of the PI3K pathway, which are currently being explored in clinical trials for ovarian cancer.

In the current study, we started with both the normal physical determination and the tissue staining profile of the axillary lymph node and ovarian cancer. Subsequently, with the application and data interpretation of the next-generation sequencing (NGS), we found mutations correlated with nucleotide metabolism pathways (such as thymine catabolism pathway, thymidine *de novo* biosynthesis pathway, and homocysteine metabolism pathway), DNA repair pathways, (such as nucleotide excision repair pathway, single-strand break repair pathway), drug resistance, HR deficiency, microsatellite stability. Among these mutation profiles, D326E is a novel missense mutation for *BTK*, Bruton’s tyrosine kinase (a nonreceptor kinase) has been reported to play a crucial role in B cell receptor (BCR) signaling. Mutations or increased expression of *BTK* are correlated in many types of B cell-derived malignancy, including chronic lymphocytic leukemia (CLL) and other B cell cancers (8). Importantly, elevated expression is not only correlated with B-cell related carcinoma. Solid tumors as breast cancer (9), pancreatic cancer (10) and other types of cancer are directly or indirectly mediated by aberrant *BTK* expression or mutations. Therefore, targeting C483S of *BTK*, the most common *BTK* mutation, has led to the development of covalent (irreversible) inhibitor and its clinical application (11). However, due to the significant role of BCR for normal B cell development and adaptive immunity, covalent (ibrutinib, acalabrutinib, and zanubrutinib) and noncovalent (pirtobrutinib and nemtabrutinib, ongoing clinical investigation) BTK inhibitor, raises drug resistance correlated with site mutations as L528W, Y223F, which is well documented elsewhere (12–14). Of note, no matter whether covalent or noncovalent BTK inhibitors target these documented mutations, combinational application of BTK inhibitor is needed to overcome BTK mutations, such as V416L, A428D, M437R, K430R and T474I, and these mutations has been experimentally demonstrated to be correlated with autophosphorylation or kinase- inactivation (12, 15). In our study, the D326E of BTK mutation, located in the SH2 domain (22), suggests an altered function that could impact downstream B-cell receptor signaling pathways, which are not traditionally associated with ovarian cancer. The mutation found in this case could lead to kinase dysfunction, possibly influencing cell signaling in a way that promotes metastatic behavior or chemoresistance. While BTK inhibitors such as ibrutinib have been developed primarily for hematologic malignancies, the presence of a BTK mutation in ovarian cancer could open avenues for novel off-label therapeutic strategies, especially if further studies support the role of BTK in solid tumor metastasis.

These findings underscore the potential for integrating comprehensive genomic profiling into routine clinical practice for ovarian cancer, especially in cases with atypical metastatic patterns. The identification of novel mutations such as *BTK* pD326E and *EPHA5* pD251E opens avenues for targeted therapies that could be tailored to individual patients’ mutational landscapes. For example, the *BTK* mutation’s role in kinase signaling suggests a need to explore off-label use of BTK inhibitors, traditionally used for hematologic malignancies, in solid tumors like ovarian cancer. Similarly, *EPHA5* mutations, which may enhance metastatic potential through impaired cell adhesion, highlight the importance of targeting ephrin receptor signaling pathways in metastatic ovarian cancer. These findings also call for the development of diagnostic panels that include *BTK* and *EPHA5* mutations, enabling earlier detection of high-risk metastatic cases. Future clinical trials focusing on these mutations could not only validate their role in tumor progression but also evaluate the efficacy of novel inhibitors or combinational therapies in improving patient outcomes.

Overall, the identification of BTK and EPHA5 mutations in this patient’s tumor highlights the expanding landscape of genetic drivers in ovarian cancer and underscores the potential for using precision medicine to address previously unidentified mutation- driven pathways. This case not only contributes to the understanding of rare metastatic patterns but also paves the way for investigating targeted therapies that may improve outcomes in patients with complex mutation profiles.

## 5 Limitations

This study has several limitations that should be acknowledged. Firstly, the follow-up period for the patient was relatively short, limiting the ability to comprehensively evaluate the long-term outcomes and recurrence patterns associated with the identified genetic mutations and the treatment approach employed. A longer follow-up would have provided a more robust assessment of the patient’s response to therapy and potential late-emerging resistance mechanisms, offering deeper insights into the prognostic implications of BTK and EPHA5 mutations in ovarian cancer metastasis. Secondly, the patient received surgery on the abdomen lesion. So, she might be at higher risk of recurrence. Thirdly, because of the financial burden, the patient did not undergo a PET-CT scan after surgery. She only performed enhanced CT and MRI scanning. Lastly, the absence of cell and animal validation experiments, such as mutation of BTK and EPHA5, molecular docking, and screening of compounds or neutralizing antibodies, were not performed due to time limit, although these experiments could provide more comprehensive data on the functional impact and potential drugs targeting these mutations. Future research incorporating these *in vitro* validations could shed further light on the biological significance of these novel mutations and their role in therapeutic resistance and metastatic progression.

## 6 Conclusion

In conclusion, this case highlights the significance of comprehensive genomic profiling and personalized drug therapy accordingly. We identified novel genetic mutations in metastatic ovarian cancer, specifically BTK (D326E) and EPHA5 (D251E), which have potential implications for targeted therapy. Moreover, these mutations together may explain the metastatic of ovarian cancer to the right axillary lymph node. These findings may enrich the understanding of the molecular mechanisms underlying lymphatic metastasis and chemoresistance.

## Data Availability

The raw data supporting the conclusions of this article will be made available by the authors, without undue reservation.
