# Supplementary figures and images for: Case report: A panorama gene profile of ovarian cancer metastasized to axillary lymph node
Source: Front Immunol. 2025 Jan 24;16:1548102. doi: 10.3389/fimmu.2025.1548102 (PMC11802514; doi:10.3389/fimmu.2025.1548102)

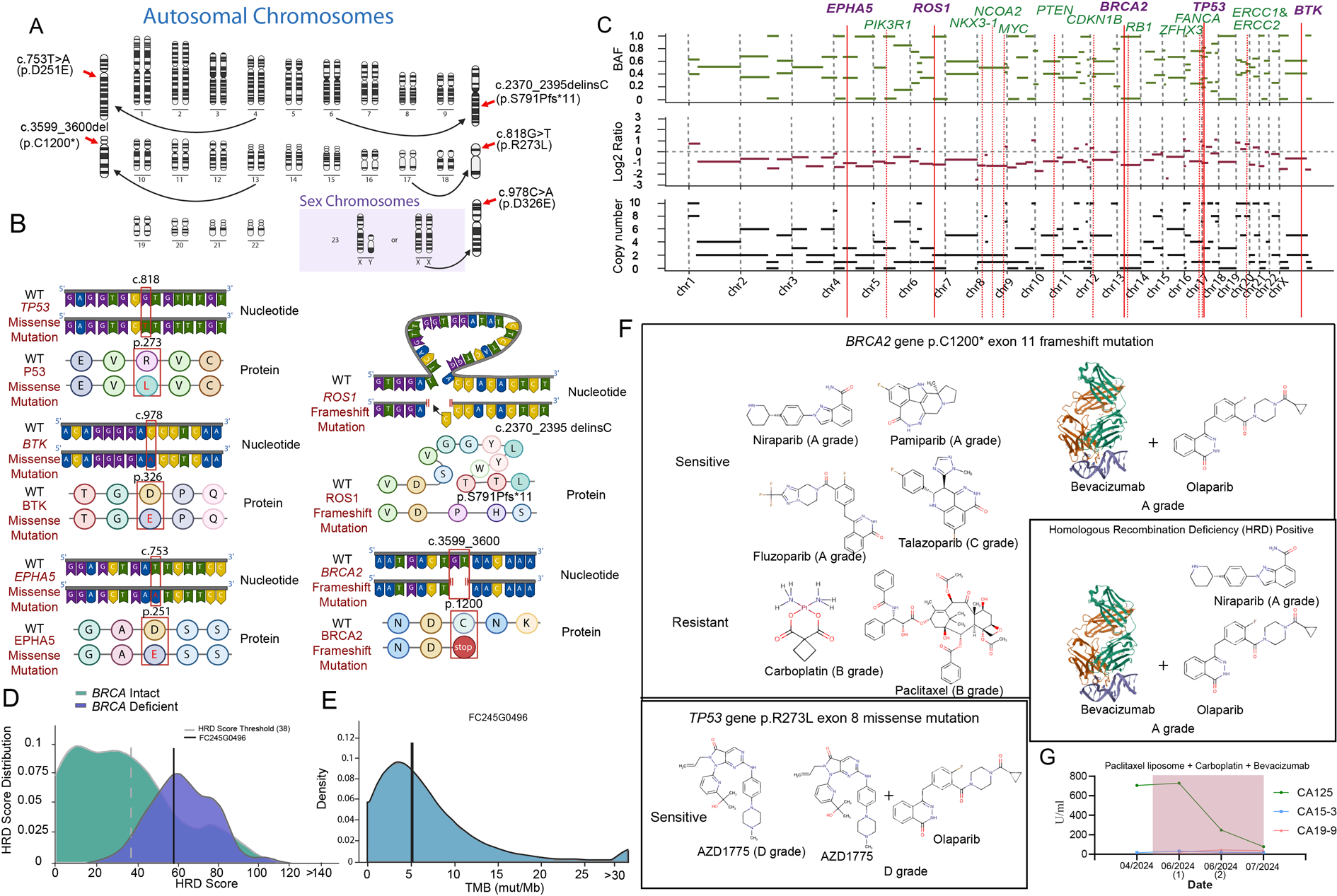

Supplement: Supplementary file 1 [file Image1.tif]

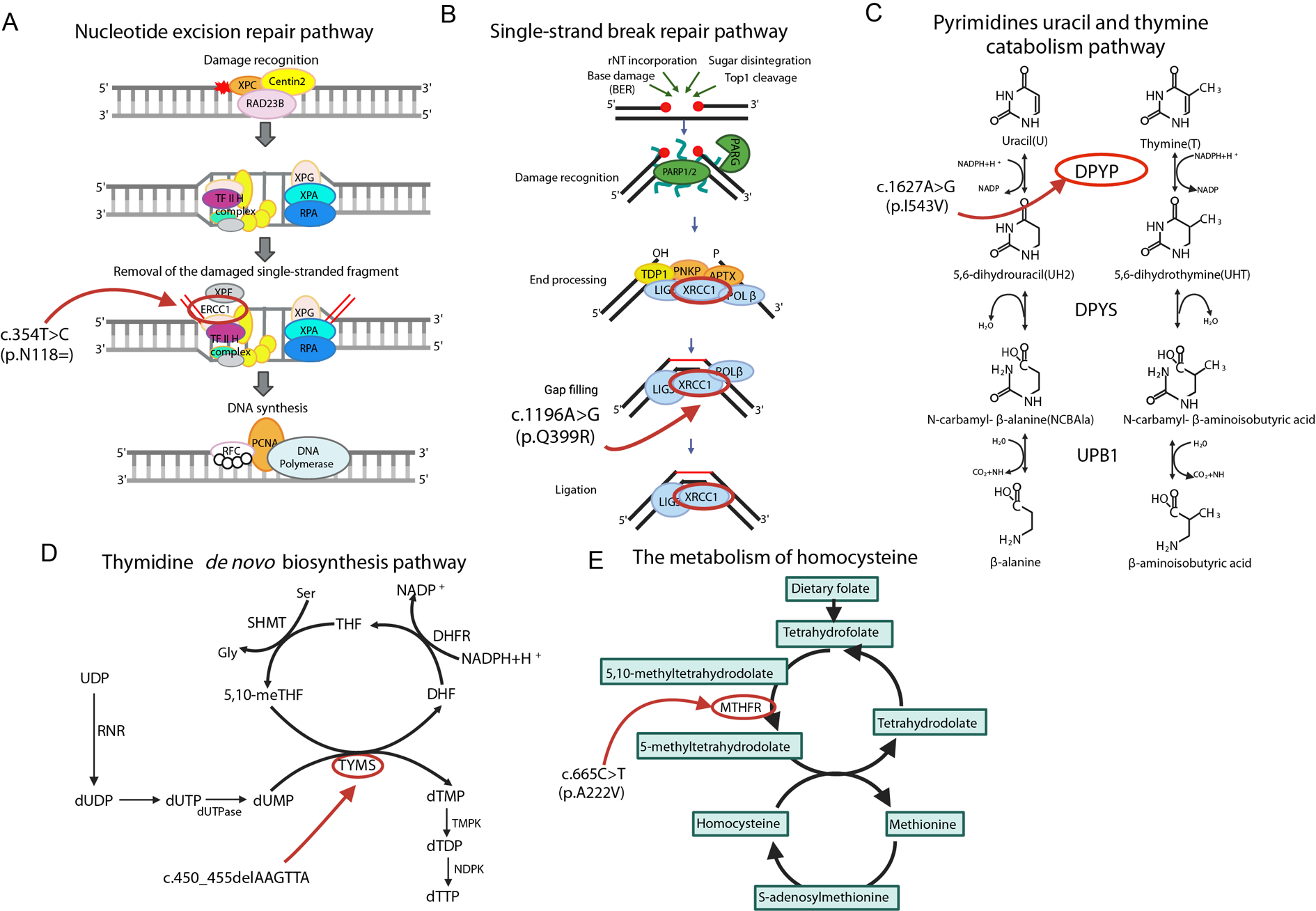

Supplement: Supplementary file 2 [file Image2.tif]

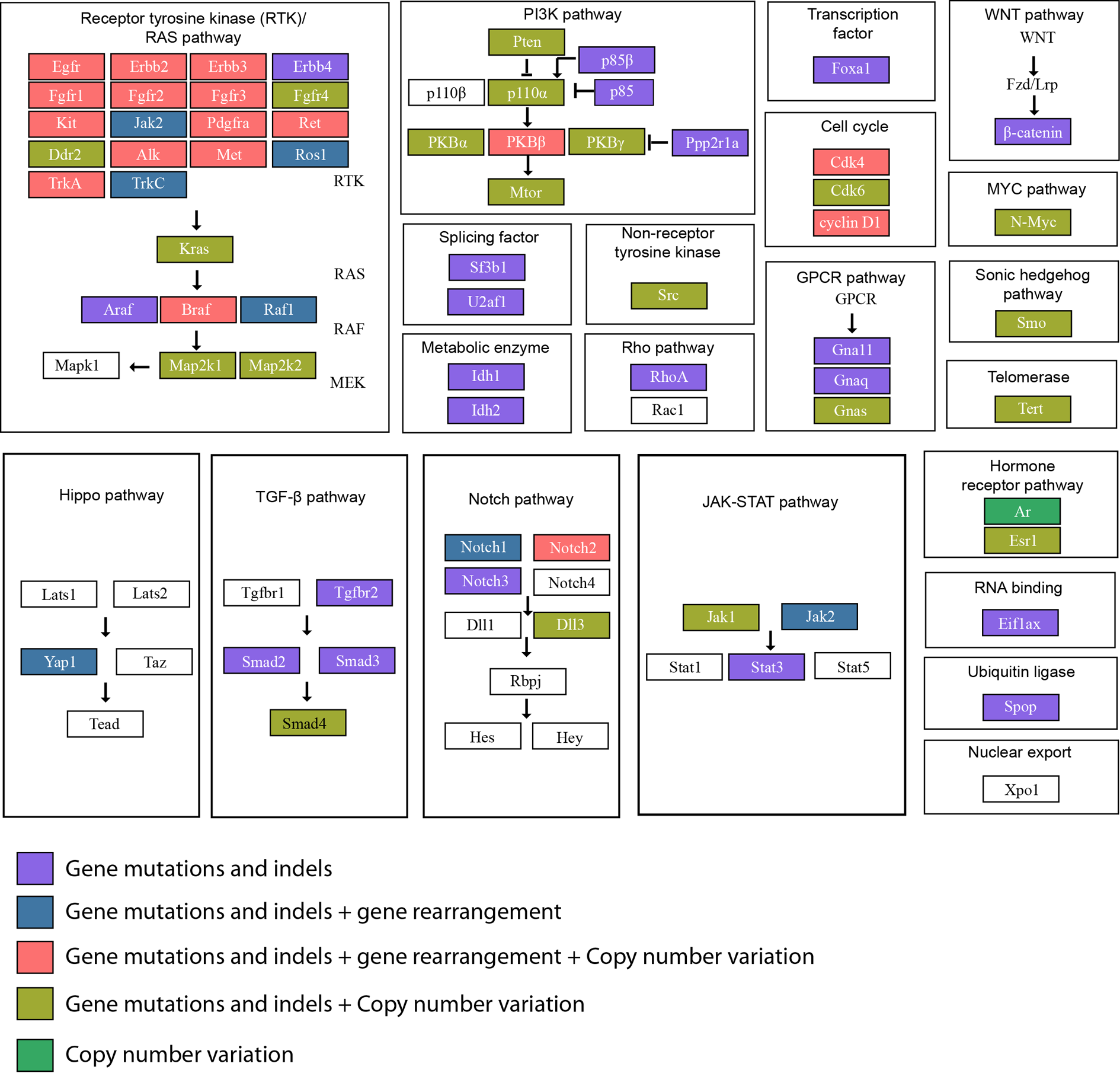

Supplement: Supplementary file 3 [file Image3.tif]

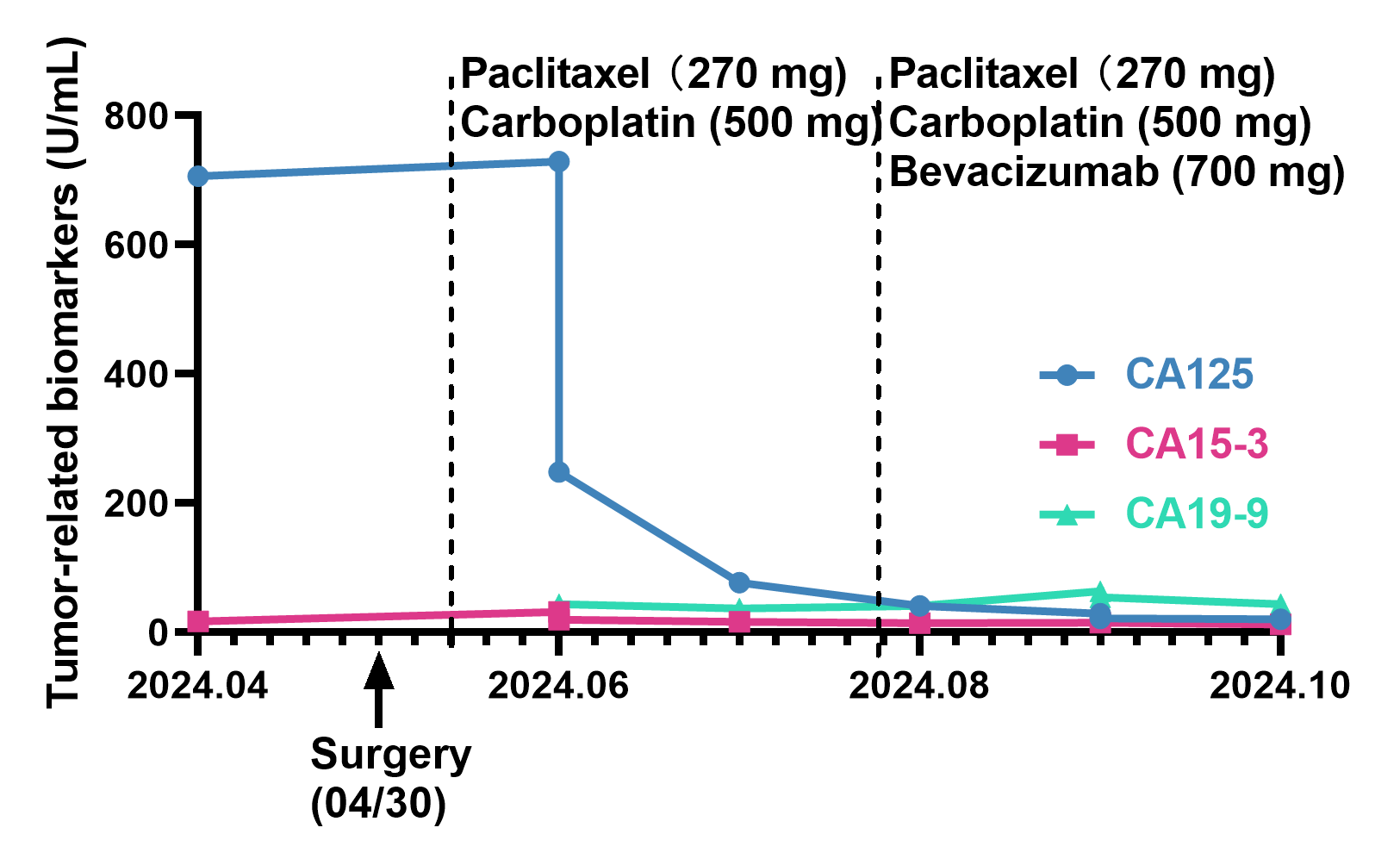

Supplement: Supplementary file 4 [file Image4.tif]

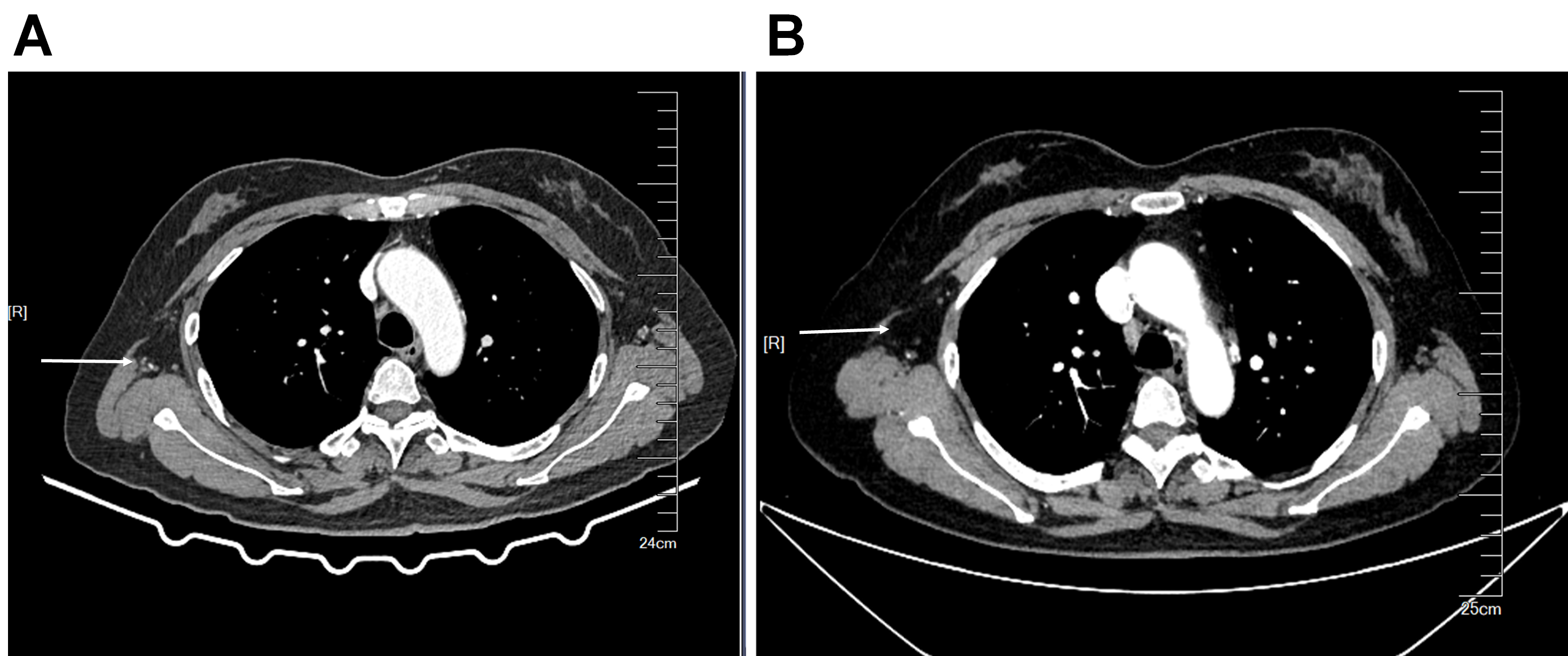

Supplement: Supplementary file 5 [file Image5.tif]
